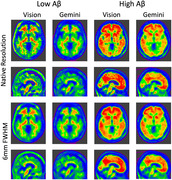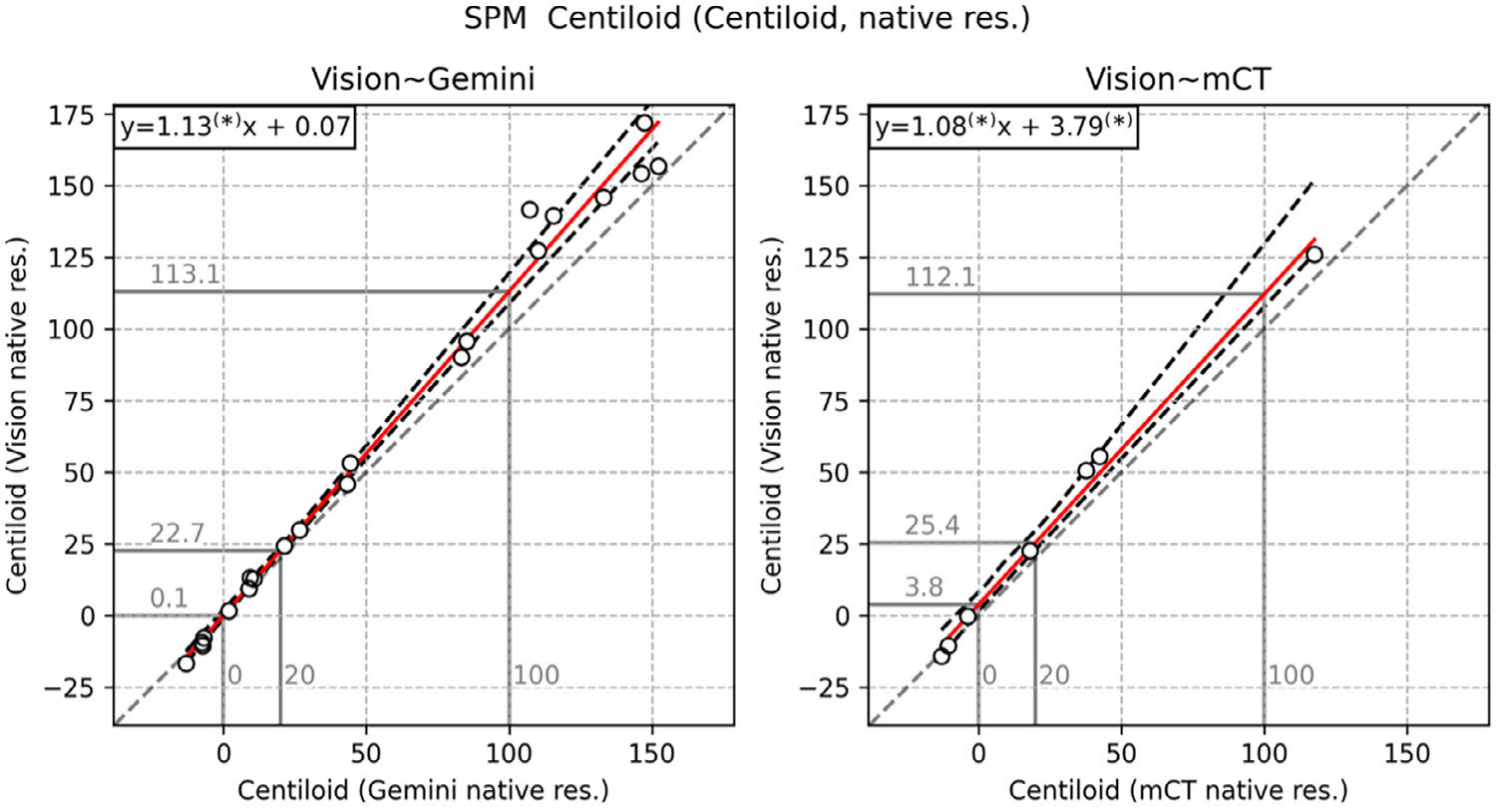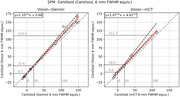# Effect of Scanner on beta amyloid quantification in a head‐to‐head comparison of 18F‐NAV4694 Centiloid measurements between Biograph Vision, Gemini and Biograph mCT

**DOI:** 10.1002/alz.087430

**Published:** 2025-01-09

**Authors:** Vincent Dore, Ashley G Gillman, Pierrick Bourgeat, Victor L Villemagne, Jurgen Fripp, Kun Huang, Robert Williams, Rosita Shishegar, Graeme O'Keefe, Timothy Cox, Shenpeng Li, Natasha Krishnadas, Azadeh Feizpour, Svetlana Bozinovski, Christopher C. Rowe

**Affiliations:** ^1^ The Australian e‐Health Research Centre, Commonwealth Scientific and Industrial Research Organisation, Melbourne, VIC Australia; ^2^ Austin Health, Melbourne, VIC Australia; ^3^ The Australian e‐Health Research Centre, Commonwealth Scientific and Industrial Research Organisation, Brisbane, QLD Australia; ^4^ University of Pittsburgh, Pittsburgh, PA USA; ^5^ National Imaging Facility, Melbourne, VIC Australia; ^6^ The University of Melbourne, Melbourne, VIC Australia; ^7^ The Florey Institute of Neuroscience and Mental Health, Melbourne, VIC Australia

## Abstract

**Background:**

New generation PET scanners achieve superior resolution and sensitivity, but the implications on beta amyloid (Aβ) quantitation are not well understood. The Centiloid (CL) scale (Klunk et al., 2015) was introduced to promote consistent Aβ burden quantification across different positron emission tomography (PET) tracers and quantification pipelines, but was not intended to control for hardware or reconstruction changes. Instead, post‐reconstruction smoothing harmonisation (Joshi, Koeppe, Fessler, 2009) is assumed to harmonise inter‐scanner Centiloid measurements. In this work, we compare paired Centiloid measurements between the Siemens Biograph Vision and one of Siemens Biograph mCT or Philips Gemini.

**Method:**

28 AIBL participants underwent MPRAGE‐MRI and ^18^F‐NAV4694 imaging on two scanners (of Vision, mCT or Gemini) within a year, reconstructed per the ADNI protocols for each scanner. Centiloid was quantified using the standard SPM framework and corrected for expected accumulation between scans. We investigated difference in Centiloid and effectiveness of PET resolution harmonisation, i.e., smoothed to 6mm‐equivalent point spread function using estimates from Hoffman scans. Statistical significance was evaluated using empirical, one‐sided, non‐parametric bootstrap estimates.

**Results:**

Figure 1 shows representative differences between the Vision and Gemini, and Figures 2,3 depict per‐scanner scatter and Bland‐Altman plots of Centiloid with and without resolution harmonisation, respectively. Even with harmonisation, Centiloid from Vision was found to be significantly higher than from Gemini (p=0.000) and from mCT (p=0.031); the difference followed a linear association with slope>1 (p=0.000 and p=0.010, respectively). The difference at 0CL was negligible, but at 100CL (Gemini) was +10.5% and at 100CL (mCT) was +11.7%. Resolution harmonisation only reduced ∼20% of scanner differences at most.

**Conclusion:**

Scanner changes led to different Centiloid quantification, with difference proportional to the participant’s Centiloid itself. The standard resolution harmonisation procedure did not correct for scanner difference. For high‐amyloid individuals, the measured difference between Vision and Gemini or mCT (> +10%) was greater than the expected annual accumulation rate and so significant for longitudinal studies. Some scanners may exaggerate measured changes in Centiloid, or longitudinal scanner changes may mask or amplify changes. The fact that the effect between scanners was found linear is promising that scanner calibration methods are possible, further investigation is warranted.